# Polyvascular Atherosclerotic Disease: A Narrative Review of the Role of Systematic Bedside Vascular Examination

**DOI:** 10.7759/cureus.116098

**Published:** 2026-09-11

**Authors:** Sai Say Han

**Affiliations:** 1 Internal Medicine, Independent Research, New York, USA

**Keywords:** arterial examination, carotid atherosclerosis, coronary artery disease, electrocardiography (ecg), generalized atherosclerosis, ischemic cerebrovascular disease, multisite arterial disease, peripheral artery disease (pad), polyvascular disease

## Abstract

Polyvascular atherosclerotic disease refers to clinically significant atherosclerosis involving two or more arterial territories and is associated with a greater risk of cardiovascular and cerebrovascular events than disease confined to a single vascular bed. Because atherosclerosis is a systemic process, the identification of disease in one territory should prompt clinicians to consider involvement elsewhere. Despite increasing reliance on advanced imaging, a systematic bedside vascular examination remains an accessible and inexpensive first step for identifying findings that may justify targeted investigation. This narrative review examines the role of clinical history and physical examination in evaluating patients with established or suspected atherosclerotic disease across multiple vascular territories. Particular attention is given to carotid auscultation, bilateral upper-extremity blood pressure and pulse assessment, cardiac evaluation, abdominal vascular examination, and examination of the femoral, popliteal, posterior tibial, and dorsalis pedis arteries, together with inspection for limb ischemic changes. The review also discusses the diagnostic limitations of individual physical findings and emphasizes that bedside examination should not be considered a substitute for objective testing. Rather, abnormal or suspicious findings can guide the selective use of ankle-brachial index (ABI) measurement, duplex ultrasonography, computed tomography angiography (CTA), magnetic resonance angiography (MRA), and appropriate cardiac investigations. A structured approach that begins with systematic clinical assessment and progresses to targeted testing may help identify clinically important multiterritory disease while avoiding indiscriminate imaging of asymptomatic vascular beds. Incorporating comprehensive vascular examination into routine assessment may therefore strengthen cardiovascular risk recognition and support more individualized investigation and management of patients with systemic atherosclerosis.

## Introduction and background

Atherosclerosis is a systemic arterial disorder that can affect multiple vascular territories simultaneously or sequentially. Although patients frequently come to medical attention because of manifestations within a single organ system, such as coronary artery disease (CAD), ischemic cerebrovascular disease, or lower-extremity peripheral artery disease (PAD), clinically relevant atherosclerosis may coexist in other vascular beds. Polyvascular disease, also termed multisite arterial disease in parts of the vascular literature, generally refers to clinically established atherosclerotic disease involving two or more major arterial territories [1-4].

The evidence base informing this narrative review includes contemporary clinical outcome studies, registries, and foundational approaches to the recognition of polyvascular disease [5-12]; diagnostic, screening, and clinical-examination studies evaluating carotid findings, inter-arm blood-pressure differences, subclavian disease, and lower-extremity vascular examination [13-20]; and disease-specific evidence addressing coronary assessment, aortic examination, renovascular disease, mesenteric ischemia, ankle-brachial index (ABI) assessment, and cardiovascular risk management [21-26].

Contemporary reviews estimate that polyvascular involvement may be present in approximately 30%-70% of selected populations with established atherosclerotic disease, although prevalence varies substantially according to the index vascular territory, disease severity, population characteristics, and methods used to identify additional disease [3,4]. 

Recognition of multiterritory disease is clinically important because cardiovascular risk increases as additional vascular beds become involved. In the European cohort of the REACH (Reduction of Atherothrombosis for Continued Health Registry), 22.8% of 19,117 symptomatic patients had polyvascular rather than single-territory disease, and patients with multiterritory involvement had a greater cardiovascular risk-factor burden and higher subsequent event rates [8]. Similarly, a secondary analysis of 13,885 participants with lower-extremity PAD in the EUCLID trial (Examining Use of Ticagrelor in Peripheral Artery Disease) demonstrated a progressive increase in major adverse cardiovascular events with additional coronary and cerebrovascular involvement [10]. Among patients with type 2 diabetes in the SAVOR-TIMI 53 (Saxagliptin Assessment of Vascular Outcomes Recorded in Patients with Diabetes Mellitus-Thrombolysis in Myocardial Infarction), cardiovascular events and mortality also increased stepwise with the number of diseased arterial beds [9]. More recently, the e-Ultimaster registry demonstrated incrementally higher mortality and coronary target-lesion failure after percutaneous coronary intervention as the number of known affected vascular territories increased [5], and a large Vascular Quality Initiative analysis found worse perioperative and long-term outcomes among patients with polyvascular disease undergoing carotid endarterectomy or lower-extremity bypass [11]. 

The concept that a patient presenting with disease in one arterial territory should undergo clinical assessment for disease elsewhere is not new. A report on the clinical approach to the polyvascular patient published in 1988 emphasized history and physical examination as the foundation of rational diagnostic evaluation, with vascular ultrasonography and angiography serving as complementary investigations [6]. In 2012, Vlachopoulos et al. similarly argued that assessment of cardiovascular risk factors, comorbid disease, and a thorough physical examination should form the initial work-up of patients suspected of having polyvascular disease [7]. Contemporary European guidance has formalized this clinical approach. The 2024 European Society of Cardiology (ESC) guidelines emphasize assessment across the arterial circulation and place vascular history and physical examination early in the diagnostic pathway, with objective vascular testing used according to the clinical presentation [1].

Because atherosclerosis is systemic, multiterritory assessment should not be equated with routine anatomical screening of asymptomatic vascular beds. In the randomized AMERICA study, systematic screening detected previously unrecognized asymptomatic multisite disease but did not significantly improve the primary clinical outcome at two years [12]. Current guidelines therefore support clinically directed testing when symptoms, physical findings, risk profile, or potential consequences for management justify further investigation [1,2,12].

Despite increasingly sophisticated vascular imaging, bedside examination remains relevant because several major arterial territories are accessible to direct clinical assessment. Neurological symptoms and carotid auscultation may raise suspicion for cerebrovascular disease; bilateral arm blood-pressure measurement and comparison of upper-extremity pulses can reveal clinically meaningful asymmetry; cardiovascular history and examination can identify indications for cardiac investigation; abdominal examination can provide clues to aortic or renovascular disease; and inspection, bruit assessment, and systematic palpation of the femoral, popliteal, posterior tibial, and dorsalis pedis arteries can identify findings associated with lower-extremity PAD [1,2]. The 2024 American College of Cardiology/American Heart Association (ACC/AHA) guideline specifically recommends a thorough lower-extremity vascular examination in individuals at increased PAD risk and identifies established atherosclerotic disease in another vascular bed as a PAD risk feature [2]. 

However, physical findings have variable diagnostic performance and should not be treated as definitive tests. A meta-analysis evaluating carotid bruits reported a sensitivity of approximately 53% and specificity of approximately 83% for severe carotid stenosis, demonstrating that the absence of a bruit cannot reliably exclude important disease [13]. The US Preventive Services Task Force consequently recommends against population screening for asymptomatic carotid stenosis and notes the poor accuracy of bruit auscultation as a screening test [14]. Conversely, peripheral pulse abnormalities and femoral bruits can meaningfully increase the probability of lower-extremity PAD, although prior studies and reviews have similarly concluded that physical examination alone cannot conclusively include or exclude PAD [16,17,19]. These limitations support using bedside examination as a method of clinical triage rather than as a replacement for objective vascular testing. 

Accordingly, this narrative review examines the evidence supporting systematic bedside vascular assessment in patients with established or suspected atherosclerotic disease. It reviews the epidemiologic and prognostic significance of polyvascular disease; evaluates clinical assessment of the cerebrovascular, upper-extremity, coronary, aortic, renal, mesenteric, and lower-extremity circulations; summarizes the diagnostic performance and limitations of important bedside findings; and discusses how examination findings can guide selective use of the ABI, duplex ultrasonography, computed tomography angiography (CTA), magnetic resonance angiography (MRA), and cardiac investigations. Particular emphasis is placed on distinguishing comprehensive multiterritory clinical assessment from routine imaging-based screening of asymptomatic arterial beds.

## Review

Literature search approach

A narrative literature search was undertaken to identify evidence relevant to polyvascular atherosclerotic disease, multisite arterial disease, and the diagnostic contribution of bedside vascular assessment. PubMed/Medical Literature Analysis and Retrieval System Online (MEDLINE) was searched using combinations of the terms “polyvascular disease,” “multisite arterial disease,” “atherosclerosis,” “vascular examination,” “physical examination,” “carotid bruit,” “peripheral pulses,” “femoral bruit,” “inter-arm blood pressure difference,” “subclavian stenosis,” “ankle-brachial index,” “renal artery stenosis,” “mesenteric ischemia,” and “vascular screening.” Contemporary guidelines from major cardiovascular and vascular societies were also reviewed. Priority was given to recent clinical practice guidelines, large prospective registries, randomized studies, systematic reviews, meta-analyses, and studies evaluating the diagnostic performance of individual components of the physical examination. Older studies were retained when they provided foundational concepts or diagnostic-accuracy data that remain relevant to contemporary practice. 

Because this was a narrative review, no prespecified systematic-review protocol, duplicate screening process, Preferred Reporting Items for Systematic Reviews and Meta-Analyses (PRISMA) flow diagram, or formal risk-of-bias assessment was used. Studies were selected by the author according to their direct relevance to the clinical question, with priority given to contemporary clinical practice guidelines, large registries, randomized studies, systematic reviews, meta-analyses, and diagnostic-accuracy studies. Older publications were retained when they provided foundational concepts or clinically relevant examination data. The final reference set was therefore intended to provide a clinically focused narrative synthesis rather than an exhaustive systematic search of all potentially eligible studies.

Polyvascular atherosclerotic disease: epidemiology and prognostic significance

Polyvascular atherosclerotic disease describes clinically established atherosclerotic involvement of at least two arterial territories and represents a particularly high-risk phenotype of systemic atherosclerosis [3,4]. The affected territories most commonly considered in major clinical studies are the coronary, cerebrovascular, and peripheral arterial circulations, although contemporary vascular practice also recognizes atherosclerotic involvement of the aorta, renal arteries, mesenteric circulation, and upper-extremity arteries [1-4]. The prevalence of polyvascular involvement varies according to the population examined, the index vascular disease, and whether additional disease is defined clinically or detected through systematic imaging. Contemporary reviews have estimated that polyvascular disease may be present in approximately 30%-70% of selected populations with known atherosclerotic disease [3]. 

Large observational datasets consistently demonstrate a gradient of cardiovascular risk according to the number of arterial territories involved. In the European cohort of the REACH Registry, 22.8% of 19,117 symptomatic patients had polyvascular disease rather than disease confined to a single arterial bed [8]. Patients with multiterritory involvement were older and had higher prevalences of smoking, hypertension, and diabetes, and experienced higher subsequent cardiovascular event rates [8]. The clinical relevance of this association extends beyond simply documenting additional anatomical disease because the number of affected territories provides information about total atherosclerotic burden and future risk. 

The EUCLID trial provided additional evidence of this risk gradient among 13,885 patients with lower-extremity PAD. Compared with isolated PAD, the adjusted risk of major adverse cardiovascular events increased when cerebrovascular disease, CAD, or both were also present. The adjusted hazard ratios were approximately 1.34 for peripheral plus cerebrovascular disease, 1.65 for peripheral plus coronary disease, and 1.99 when all three major vascular territories were involved [10]. These findings demonstrate a stepwise increase in cardiovascular risk rather than a simple binary distinction between the presence and absence of polyvascular disease. 

A similar pattern has been demonstrated in patients with type 2 diabetes mellitus. In a secondary analysis of SAVOR-TIMI 53 involving 16,492 participants, the adjusted hazard ratio for cardiovascular death, myocardial infarction, or ischemic stroke increased from 1.95 among patients with one diseased vascular bed to 3.54 with two diseased beds and 4.64 with three diseased beds compared with participants without established atherosclerosis [9]. Overall mortality also increased progressively with the number of territories involved [9]. 

Contemporary interventional populations demonstrate the same relationship. In the e-Ultimaster registry of 37,198 patients undergoing percutaneous coronary intervention, target-lesion failure increased from 3.16% in patients without known prior vascular disease to 4.44% with single-territory disease and 6.42% with multisite disease. All-cause mortality similarly increased from 2.22% to 3.28% and 5.29%, respectively [5]. A 2024 analysis of the Vascular Quality Initiative found polyvascular disease in approximately 47% of patients undergoing carotid endarterectomy or lower-extremity bypass. Perioperative myocardial infarction or death and five-year mortality increased progressively as additional vascular beds became involved [11]. 

Collectively, these findings support the view that atherosclerosis should not be conceptualized exclusively according to the organ responsible for the presenting event. The recognition of additional vascular involvement identifies a population with greater systemic atherosclerotic burden and substantially higher cardiovascular risk [3-5,8-11].

Historical and contemporary basis for multiterritory clinical assessment

The concept of examining patients for disease beyond the initially affected vascular territory predates modern cross-sectional imaging. In 1988, Zicot described history and physical examination as foundational elements in the assessment of the polyvascular patient and emphasized that complementary vascular investigations should subsequently be selected according to the individual clinical context [6]. The article specifically discussed ultrasonography as an extension of physical examination across the supra-aortic vessels, aorta and its branches, and limb arteries [6]. 

Vlachopoulos et al. revisited the concept in 2012 and described risk-factor assessment, known comorbid disease, and a thorough physical examination as mandatory components of the initial evaluation of suspected polyvascular disease [7]. They also discussed duplex ultrasonography, CTA, and MRA as subsequent methods for identifying additional vascular involvement. Importantly, the authors highlighted a question that remains clinically relevant: whether identifying otherwise asymptomatic disease in additional arterial territories necessarily improves patient outcomes [7]. 

Contemporary ESC guidance incorporates a similar clinical philosophy. The 2024 ESC guidelines integrate peripheral arterial and aortic disease within a unified framework and emphasize clinical history and physical examination as early components of vascular assessment [1]. The 2024 ACC/AHA lower-extremity PAD guideline likewise identifies established atherosclerotic disease in another vascular bed as an important PAD risk feature and recommends history and physical examination before objective diagnostic testing [2].

The modern rationale for bedside multiterritory assessment is therefore not that physical examination can identify every clinically significant lesion. Rather, examination provides an initial method for identifying symptoms and signs that can modify pretest probability and determine whether objective physiological or anatomical testing is warranted.

Cerebrovascular and carotid assessment

Assessment of the cerebrovascular territory begins primarily with the clinical history. Previous stroke or transient ischemic attack, focal motor or sensory symptoms, speech disturbance, and transient monocular visual loss provide substantially more clinically actionable information than isolated neck auscultation. Carotid auscultation nevertheless remains a traditional component of comprehensive vascular examination because an audible bruit may identify underlying turbulent arterial flow and increase suspicion for carotid atherosclerosis [1,13].

The diagnostic limitations of carotid bruits are well established. McColgan et al. performed a meta-analysis of 26 studies involving 15,117 carotid arteries. For clinically relevant carotid stenosis (>70%), the pooled sensitivity of a carotid bruit was 53% (95% CI, 50%-55%) and specificity was 83% (95% CI, 82%-84%), with an area under the summary receiver-operating characteristic curve of 0.73 [13]. The meta-analysis also reported significant between-study heterogeneity across stenosis groups, further supporting cautious interpretation of the pooled diagnostic estimates [13]. The relatively low sensitivity is clinically important because substantial carotid stenosis may be present in the absence of an audible bruit. Conversely, the presence of a bruit does not reliably determine either the degree or exact location of stenosis [13]. 

This distinction is particularly important when bedside assessment is considered in the context of screening. The US Preventive Services Task Force recommends against screening the general asymptomatic adult population for carotid artery stenosis and specifically notes that auscultation for carotid bruits has poor accuracy as a screening test [14]. This recommendation applies to asymptomatic population screening and should not be interpreted as eliminating carotid auscultation from the examination of a patient with established systemic vascular disease or relevant neurological symptoms. Instead, an abnormal clinical finding should be interpreted within the overall clinical context and, when appropriate, followed by carotid duplex ultrasonography, CTA, or MRA [1,14]. 

Thus, carotid examination demonstrates a principle applicable throughout this review: the value of an abnormal physical sign may lie in raising suspicion, whereas the absence of that sign generally cannot exclude disease.

Upper-extremity and subclavian arterial assessment

The upper extremities represent an accessible but sometimes overlooked component of the vascular examination. History should include exertional arm fatigue or discomfort, asymmetric exercise tolerance, and neurological symptoms associated with upper-extremity activity when subclavian steal physiology is clinically suspected. Examination includes comparison of radial and brachial pulses and measurement of blood pressure in both arms [1].

Inter-arm systolic blood pressure difference has been studied both as a marker of localized subclavian disease and as a measure of generalized cardiovascular risk. A 2012 systematic review and meta-analysis found that a systolic inter-arm difference of at least 10 mmHg was strongly associated with angiographically demonstrated subclavian stenosis, while larger inter-arm differences were associated with peripheral vascular disease and increased cardiovascular and all-cause mortality [20]. 

The INTERPRESS-IPD (Inter-arm Blood Pressure Difference - Individual Participant Data) Collaboration subsequently pooled individual participant data from 24 studies involving 53,827 participants. Increasing systolic inter-arm difference was independently associated with cardiovascular events, cardiovascular mortality, and all-cause mortality, with risk increasing progressively rather than appearing only above a single threshold [15]. The investigators proposed 10 mmHg as an upper limit of normal for systolic inter-arm difference in routine cardiovascular assessment [15]. 

The association with subclavian arterial disease is also supported by population data. Shadman et al. evaluated 4,223 participants and defined subclavian stenosis by an inter-arm systolic pressure difference of at least 15 mmHg. The prevalence was approximately 1.9% in community cohorts and 7.1% in clinical cohorts, and subclavian stenosis was strongly associated with concomitant PAD [18]. 

These findings support bilateral arm blood-pressure measurement and pulse comparison as low-cost components of a broader vascular assessment. However, inter-arm difference is not sufficiently sensitive to exclude subclavian disease when absent, and persistent asymmetry should prompt interpretation in conjunction with symptoms and other examination findings rather than automatic vascular imaging.

Coronary and cardiac assessment

The coronary circulation differs from peripheral arterial territories because obstructive coronary atherosclerosis cannot be reliably diagnosed through palpation or auscultation. The bedside contribution therefore relies primarily on cardiovascular history, assessment of functional capacity, examination for associated cardiac disease, and appropriate use of electrocardiography when symptoms warrant further evaluation.

Patients with established extracoronary atherosclerosis have a higher prevalence of coronary disease and greater cardiovascular risk than individuals without systemic vascular disease [3-5,8-11]. Consequently, a multiterritory history should include exertional chest pressure or discomfort, exertional dyspnea, reduction in exercise tolerance, and previous myocardial infarction or coronary revascularization. However, the mere presence of extracoronary atherosclerosis does not establish occult obstructive CAD or justify routine coronary angiography in asymptomatic individuals.

Contemporary chest-pain guidance emphasizes that diagnosis of ischemic syndromes requires integration of history, physical examination, electrocardiography, biomarkers when appropriate, and risk-directed additional testing rather than reliance on any single clinical finding [21]. A normal resting electrocardiogram also cannot by itself exclude acute coronary syndrome [21]. 

Accordingly, the role of cardiac assessment within a polyvascular examination is principally to identify symptoms or clinical findings that warrant guideline-directed cardiac evaluation. This is distinct from performing routine anatomical coronary screening solely because disease has been documented in another vascular territory.

Aortic and abdominal vascular assessment

Abdominal examination provides an opportunity to assess for clinically apparent aortic abnormalities, although its diagnostic performance depends heavily on aneurysm size and patient characteristics. The most extensively studied physical maneuver is directed palpation of the abdominal aorta.

In a Rational Clinical Examination review pooling 15 studies, the sensitivity of abdominal palpation increased with aneurysm diameter: approximately 29% for abdominal aortic aneurysms measuring 3.0-3.9 cm, 50% for aneurysms measuring 4.0-4.9 cm, and 76% for aneurysms measuring at least 5.0 cm [22]. A palpable widened aortic pulsation therefore substantially increases suspicion for an abdominal aortic aneurysm, but a normal abdominal examination cannot reliably exclude one [22]. Obesity may further reduce examination sensitivity. 

The practical implication is similar to that for carotid examination. A positive finding may be clinically useful, particularly in a patient with systemic atherosclerosis, but physical examination should not substitute for guideline-directed ultrasonographic aneurysm screening when a patient independently meets established screening criteria [1].

Renal arterial assessment

Atherosclerotic renal artery stenosis is frequently associated with a broader systemic atherosclerotic phenotype and may coexist with coronary, carotid, and lower-extremity arterial disease [1,23]. The bedside examination has limited ability to establish the diagnosis, but certain clinical patterns can raise suspicion for renovascular disease.

These include resistant or rapidly worsening hypertension, deterioration in kidney function in an appropriate clinical context, recurrent unexplained pulmonary edema, and abdominal or flank vascular bruits [23]. An abdominal bruit can therefore contribute to the overall clinical picture but is neither sufficiently sensitive nor sufficiently specific to confirm or exclude renal artery stenosis. Older studies have demonstrated an association between abdominal bruits and renal artery stenosis while also showing that important stenoses may remain clinically silent. 

The AHA scientific statement on renovascular disease emphasizes that patient selection for further evaluation and potential intervention should be based on the complete clinical syndrome rather than simply the anatomical presence of renal artery narrowing [23]. Once clinical suspicion is sufficient, duplex ultrasonography, CTA, or MRA can provide objective evaluation. 

Thus, renal vascular assessment reinforces the importance of pattern recognition: a bruit alone is weak evidence, but a bruit occurring alongside resistant hypertension, renal dysfunction, or recurrent pulmonary edema carries substantially different clinical significance.

Mesenteric arterial assessment

Mesenteric atherosclerosis provides an example in which clinical history is considerably more important than routine physical examination. Chronic mesenteric ischemia typically occurs when postprandial intestinal blood-flow requirements cannot be met because of significant mesenteric arterial occlusive disease. Classic symptoms include postprandial abdominal pain, food avoidance or fear of eating, and progressive weight loss [24]. 

An abdominal vascular bruit may occasionally be present but is neither sufficiently sensitive nor specific to diagnose mesenteric ischemia. Therefore, routine abdominal auscultation should not be interpreted as a screening test for mesenteric stenosis. Instead, a compatible symptom complex in an individual with extensive atherosclerotic disease should increase suspicion and prompt appropriate imaging. Society for Vascular Surgery guidelines identify CTA as an important component of diagnostic evaluation when chronic mesenteric ischemia is suspected [24]. 

This territory illustrates why a “systematic examination” should not be equated with a fixed series of auscultatory maneuvers. In some vascular beds, the history provides substantially greater diagnostic information than the physical sign.

Lower-extremity arterial examination

The lower extremities offer the greatest opportunity for direct bedside assessment because multiple arterial segments can be palpated and ischemic tissue changes can be inspected directly. The 2024 ACC/AHA guideline recommends a thorough lower-extremity vascular examination in patients at increased risk of PAD and identifies known atherosclerotic disease in another vascular bed as a major risk feature [2].

History should assess both classic and atypical manifestations. Typical intermittent claudication consists of reproducible exertional discomfort involving the buttock, thigh, calf, or foot that improves with rest. However, many patients with objectively confirmed PAD do not report classic claudication. Walking limitation, exertional leg fatigue or weakness, ischemic rest pain, and nonhealing wounds may provide important diagnostic clues [2]. 

Physical examination should include inspection of the legs and feet and bilateral palpation of the femoral, popliteal, posterior tibial, and dorsalis pedis arteries [2]. Pulse findings are more informative when compared bilaterally and considered in combination rather than interpreted in isolation. Femoral auscultation may identify proximal turbulent flow, while inspection may demonstrate nonhealing ulcers, pallor on elevation, dependent rubor, asymmetric trophic changes, or tissue loss [2].

A systematic review by Khan et al. evaluated 17 studies comparing elements of the clinical examination with ABI, duplex ultrasonography, or angiography. Abnormal peripheral pulses and femoral bruits increased the probability of PAD, but no single physical examination finding provided sufficient sensitivity to exclude disease reliably [16]. 

Armstrong et al. subsequently evaluated 1,236 patients undergoing complete peripheral vascular examination and ABI assessment. Combining pedal pulse examination with femoral bruit assessment produced a sensitivity of 58.2%, specificity of 98.3%, negative predictive value of 94.9%, positive predictive value of 81.1%, and overall accuracy of 93.8% for PAD defined by ABI ≤0.90 [17]. The high specificity supports the clinical value of a clearly abnormal examination, whereas the considerably lower sensitivity again demonstrates that a normal examination does not reliably exclude disease [17]. A subsequent 2018 diagnostic-accuracy study in a high-risk primary-care population found that defining an abnormal examination as absence of either the dorsalis pedis or posterior tibial pulse yielded a sensitivity of 86.8% (95% CI, 74.8%-98.9%) and specificity of 82.7% (95% CI, 78.0%-87.3%) for PAD compared with Doppler ABI assessment [27]. These findings provide more recent support for the diagnostic value of pedal pulse examination while also illustrating that performance varies according to the population and examination definition.

Pulse examination also requires anatomical awareness. The dorsalis pedis pulse may be congenitally absent or difficult to palpate in some individuals, and examiner agreement is generally better for distinguishing clearly present from clearly absent pulses than for assigning intermediate grades [2]. For this reason, pulse findings should be interpreted collectively with symptoms, skin findings, femoral bruits, and objective physiological testing.

The ankle-brachial index as the transition from bedside examination to objective testing

The ABI represents an important bridge between clinical examination and anatomical vascular imaging. It provides objective physiological evidence of lower-extremity arterial obstruction without immediately exposing the patient to contrast, radiation, or invasive angiography.

The 2024 ACC/AHA guideline recommends resting ABI testing when the history or physical examination suggests PAD [2]. A resting ABI ≤0.90 is considered abnormal, whereas an ABI >1.40 suggests noncompressible arteries and may require alternative physiological testing such as toe-brachial index assessment [2]. Exercise ABI can be useful in patients with exertional symptoms despite a normal or borderline resting ABI.

The prognostic importance of ABI extends beyond the lower limb. The ABI Collaboration pooled data from multiple population cohorts and demonstrated that a low ABI independently predicted cardiovascular events and mortality and improved cardiovascular risk classification beyond the Framingham Risk Score [25]. Thus, ABI is not merely a test of limb perfusion; it also provides information about systemic atherosclerotic burden [25]. 

However, ABI itself should not be interpreted as justification for indiscriminate downstream imaging. Duplex ultrasonography, CTA, MRA, and catheter angiography are most informative when lesion anatomy is required for diagnosis, treatment planning, or revascularization decisions [1,2].

Systematic clinical assessment versus indiscriminate imaging

One of the most important distinctions in the management of polyvascular disease is the difference between systematically considering other vascular territories and systematically imaging them.

The presence of disease in one arterial territory justifies increased awareness of symptoms and signs arising from others because multiterritory involvement is common and prognostically important [3-5,8-11]. This does not mean that every patient with established atherosclerosis should undergo CTA, MRA, duplex ultrasonography, or other imaging of every asymptomatic vascular bed.

The AMERICA randomized study directly explored this issue. Among 521 high-risk patients with severe CAD, systematic screening identified previously unrecognized asymptomatic multisite arterial disease in 21.7% of patients assigned to the proactive strategy [12]. However, systematic detection combined with intensified secondary prevention did not significantly improve the primary clinical outcome at two years compared with conventional care [12]. 

These findings do not imply that identification of polyvascular disease is unimportant. Instead, they suggest that anatomical detection alone does not necessarily produce better outcomes when the information does not materially change management. A clinically directed strategy therefore offers a rational middle ground: obtain a multiterritory vascular history, perform an appropriately comprehensive bedside examination, and proceed to objective physiological testing or imaging when findings are abnormal, symptoms are suggestive, or the result is expected to influence management [1,2,12].

Clinical implications of detecting polyvascular disease

Recognition of polyvascular disease can influence cardiovascular risk stratification even when it does not lead to immediate vascular intervention. Patients with multiple diseased vascular territories consistently experience higher rates of cardiovascular death, myocardial infarction, stroke, limb events, and overall mortality than patients with single-territory disease [5,8-11].

The COMPASS (Cardiovascular Outcomes for People Using Anticoagulation Strategies) trial population illustrates the therapeutic relevance of this higher-risk phenotype. In a prespecified risk analysis, disease involving at least two vascular beds identified patients at particularly high risk for recurrent vascular events. Among higher-risk patients, the absolute benefit associated with intensified antithrombotic therapy was greater than among patients without these high-risk features [26]. Treatment decisions must nevertheless remain individualized because intensified antithrombotic therapy also carries bleeding risk and should follow applicable disease-specific recommendations rather than the presence of polyvascular disease alone [26]. 

Detection of additional disease may also prompt closer attention to modifiable risk factors, including smoking, hypertension, diabetes, dyslipidemia, physical inactivity, and adherence to preventive therapy. The clinical value of recognizing polyvascular disease is therefore broader than identifying lesions for revascularization; it may alter the clinician's perception of the patient's overall cardiovascular risk.

Integrating bedside examination with modern vascular diagnostics

The evidence reviewed demonstrates an apparent paradox. Polyvascular atherosclerotic disease is common and strongly associated with poor cardiovascular outcomes, yet individual bedside examination findings frequently have inadequate sensitivity to detect all clinically important disease. This does not make physical examination obsolete. Instead, it defines its appropriate role.

Carotid bruit auscultation, peripheral pulse assessment, femoral bruit detection, bilateral arm blood-pressure measurement, abdominal palpation, and inspection for ischemic limb changes each provide different amounts of diagnostic information. Across most arterial territories, abnormal findings have greater value for increasing suspicion than normal findings have for excluding disease [13,15-18,20,22]. The bedside examination therefore functions most effectively as a risk-stratification and triage layer between the history and more definitive physiological or anatomical testing.

This approach may also reduce territorial tunnel vision, in which a patient presenting with stroke, CAD, or lower-extremity PAD is evaluated only within the symptomatic organ system despite the systemic nature of atherosclerosis. The value of multiterritory bedside assessment is therefore to broaden clinical attention while interpreting individual findings within their appropriate vascular context. Examination findings should be integrated with the patient’s pretest probability and used to guide territory-specific, guideline-directed objective testing when appropriate [1,2].

The major components of a practical multiterritory vascular assessment and the corresponding targeted investigations are summarized in Table 1.

**Table 1:** Systematic bedside assessment of major arterial territories in patients with atherosclerotic disease CAD: coronary artery disease; CTA: computed tomography angiography; MRA: magnetic resonance angiography; ABI: ankle-brachial index

Vascular territory	Key history	Bedside assessment	Findings raising suspicion	Targeted next step
Cerebrovascular/carotid	TIA or stroke symptoms, transient monocular visual loss, focal weakness, sensory disturbance, or speech disturbance [1,14]	Focused neurologic assessment; carotid auscultation [1,13]	Focal neurologic symptoms or carotid bruit [13,14]	Carotid duplex ultrasonography; CTA/MRA when clinically indicated [1,14]
Upper-extremity/subclavian	Arm claudication, exertional fatigue, or neurologic symptoms associated with arm use [1,18]	Blood pressure in both arms; comparison of brachial and radial pulses [1,15,20]	Persistent inter-arm BP difference or pulse asymmetry [15,18,20 ]	Duplex ultrasonography; CTA/MRA if further anatomical definition is required [1,18]
Coronary/cardiac	Exertional chest discomfort, dyspnea, reduced exercise tolerance, or known CAD [21]	Cardiovascular examination; assessment of pulse and hemodynamic status [21]	Symptoms or findings suggesting ischemic or structural cardiac disease [21]	ECG and guideline-directed cardiac testing based on clinical presentation [21]
Aortic/abdominal	Abdominal or back symptoms; known aneurysmal or systemic vascular disease [1,22]	Abdominal palpation and vascular examination [1,22]	Palpable widened aortic pulsation or compatible clinical presentation [22]	Ultrasonography or CTA according to suspected pathology [1,22]
Renal	Resistant or rapidly worsening hypertension, deterioration in renal function, or recurrent unexplained pulmonary edema [23]	Blood-pressure assessment; abdominal/flank auscultation [1,23]	Compatible clinical syndrome with an abdominal or renal-area bruit [23]	Renal artery duplex ultrasonography, CTA, or MRA when clinically warranted [23]
Mesenteric	Postprandial abdominal pain, food avoidance, weight loss, or symptoms concerning for acute intestinal ischemia [24]	Abdominal examination; vascular auscultation when appropriate [1,24]	Compatible ischemic symptom pattern with or without an abdominal bruit [24]	CTA when mesenteric ischemia is clinically suspected [24]
Lower extremity	Claudication, walking impairment, ischemic rest pain, or nonhealing wounds [2,16,17]	Inspect legs and feet; palpate femoral, popliteal, posterior tibial, and dorsalis pedis pulses; auscultate femoral arteries [2,16,17]	Pulse deficit, femoral bruit, pallor, dependent rubor, ulceration, or tissue loss [2,16,17]	Resting ABI first; duplex ultrasonography and CTA/MRA selectively when clinically indicated [2,25]

The findings summarized in Table 1 should be interpreted as components of clinical risk assessment rather than definitive diagnostic criteria; abnormal findings can increase suspicion for vascular disease, whereas normal findings do not consistently exclude clinically important disease [1,2,13,16,17].

Limitations and evidence gaps

The evidence supporting individual components of bedside vascular examination is heterogeneous. Many diagnostic-accuracy studies evaluating carotid bruits, peripheral pulses, femoral bruits, and abdominal palpation were conducted before widespread use of contemporary vascular imaging and intensive preventive therapies [13,16,17,22]. Diagnostic performance can also be influenced by examiner experience, arterial anatomy, disease severity, body habitus, age, and comorbid conditions. In particular, pulse grading and bruit detection are subject to interobserver variability. These limitations help explain why abnormal physical findings may increase suspicion for vascular disease, whereas normal findings generally cannot exclude clinically important atherosclerosis [13,16,17].

Because this is a single-author narrative review without a prespecified systematic selection protocol or independent duplicate screening, study selection and interpretation may be subject to selection and citation bias. The literature was selected according to clinical relevance rather than through a systematic screening process; therefore, this review should be interpreted as a narrative synthesis and is not intended to provide the exhaustive search and reproducible methodology expected of a systematic review.

Another important limitation is that most available studies examine individual signs or individual vascular territories rather than evaluating a standardized multiterritory bedside examination as a unified diagnostic strategy. Although the adverse prognosis associated with polyvascular disease is consistently demonstrated across large registries and clinical trials [5,8-11], this does not establish that systematic examination of every accessible arterial territory improves cardiovascular outcomes. Similarly, the randomized AMERICA study demonstrated that identifying additional asymptomatic arterial disease through systematic screening did not significantly improve the primary clinical outcome at two years [12]. Therefore, the prognostic importance of polyvascular disease should be distinguished from evidence supporting routine anatomical screening of asymptomatic vascular beds.

There is also limited evidence defining the optimal threshold at which an abnormal bedside finding should trigger additional physiological testing or vascular imaging. Current ESC and ACC/AHA guidelines support history and physical examination as early components of vascular assessment and recommend objective testing when clinical findings or symptoms raise sufficient suspicion [1,2]. However, the diagnostic pathway differs according to the vascular territory, and no single standardized head-to-toe examination protocol has been prospectively validated across patients with coronary, cerebrovascular, and peripheral atherosclerotic disease. Accordingly, this review does not propose a universal bedside threshold or cross-territory testing algorithm; decisions regarding further physiological or anatomical testing should remain territory-specific and should integrate symptoms, examination findings, pretest probability, and applicable guideline recommendations.

Future prospective studies could compare a standardized multiterritory bedside vascular assessment with usual clinical care. Relevant outcomes should include detection of previously unrecognized clinically actionable disease, appropriate use of ABI and vascular imaging, changes in preventive treatment, referrals for vascular intervention, diagnostic yield, healthcare utilization, cost-effectiveness, and cardiovascular outcomes. Such studies would clarify whether formal incorporation of a structured multiterritory examination provides measurable benefit beyond current disease-specific approaches.

## Conclusions

Polyvascular atherosclerotic disease reflects the systemic distribution of atherosclerosis and is associated with greater cardiovascular risk than disease confined to a single arterial territory. The reviewed literature shows that clinical history and bedside vascular examination can identify symptoms, pulse abnormalities, bruits, blood-pressure asymmetry, and ischemic changes that raise suspicion for additional vascular involvement. However, individual physical findings have limited sensitivity, and a normal examination does not reliably exclude clinically important disease.

Bedside vascular assessment is therefore best used as an initial clinical assessment that informs the selective use of objective testing, including the ABI, duplex ultrasonography, CTA, MRA, and appropriate cardiac investigations. The available evidence supports distinguishing systematic multiterritory clinical assessment from routine imaging of asymptomatic vascular beds.
